# Cancer Survivorship After COVID-19: Psychological Burden, Symptom Experience, and Social Support

**DOI:** 10.3390/medicina62081475

**Published:** 2026-07-30

**Authors:** Anna Tsiakiri, Pinelopi Vlotinou, Konstantinos Tzanas, Despoina Chrisostomidou, Sevasti Chatzifotiou, Maria Lavdaniti, Evangeli Bista

**Affiliations:** 1 Department of Neurology, School of Medicine, Democritus University of Thrace, 68100 Alexandroupolis, Greece; 2 Department of Occupational Therapy, University of West Attica, 12243 Athens, Greece; pvlotinou@uniwa.gr; 3 Cancer Patient Guidance Center (KAPA3), 11141 Athens, Greece; ktzanas96@gmail.com (K.T.); education@kapa3.gr (D.C.); libista@yahoo.gr (E.B.); 4 Department of Social Work, Democritus University of Thrace, 69100 Komotini, Greece; schatzif@sw.duth.gr; 5 Nursing Department, International Hellenic University, Sindos, 57400 Thessaloniki, Greece; maria_lavdaniti@yahoo.gr

**Keywords:** cancer survivors, psychological distress, quality of life, social support, depression, anxiety, stress, oncology, COVID-19, psycho-oncology

## Abstract

*Background and objectives*: Cancer survivors continue to face significant psychological and quality-of-life challenges following the COVID-19 pandemic. This study aimed to investigate levels of depression, anxiety, stress, quality of life, and perceived social support among patients with cancer during the post-COVID-19 period and to examine their interrelationships. *Materials and Methods*: A cross-sectional study was conducted among 162 Greek adults with cancer. Participants completed questionnaires assessing psychological distress (Depression Anxiety Stress Scale-21), health-related quality of life (EORTC QLQ-C30), and perceived social support. Descriptive statistics, factor analysis, group comparisons, and correlation analyses were performed. *Results*: High levels of psychological distress were observed, with 32.1% of participants reporting severe or extremely severe depression, 40.1% severe or extremely severe anxiety, and 29.0% severe or extremely severe stress. Quality of life was moderate, with emotional and social functioning being the most impaired domains, while fatigue, insomnia, and financial difficulties were the most prominent symptoms. Depression, anxiety, and stress were strongly associated with poorer quality of life and greater symptom burden. Higher perceived social support, particularly from family and friends, was associated with better quality of life and lower psychological distress. *Conclusions*: These findings highlight the need for comprehensive oncology care that incorporates routine psychological assessment and psychosocial support to improve cancer survivorship outcomes.

## Simple Summary

Cancer affects not only physical health but also emotional well-being, daily functioning, and social relationships. The COVID-19 pandemic created additional challenges for people living with cancer by disrupting healthcare services, increasing uncertainty, and reducing opportunities for social interaction and support. This study examined psychological distress, quality of life, and perceived social support among Greek adults with cancer during the post-pandemic period. The findings showed that many patients continued to experience high levels of anxiety, depression, and stress, together with substantial symptom burden and reduced quality of life. Psychological distress was strongly associated with poorer functioning and greater symptom severity. In contrast, support from family members and friends was linked to better quality of life and lower emotional distress. These results highlight the importance of integrating routine psychological screening and psychosocial support into cancer care and survivorship programs to improve the overall well-being of people living with cancer.

## 1. Introduction

Cancer remains one of the leading causes of morbidity and mortality worldwide and constitutes a major global public health challenge, with substantial consequences for patients’ physical, psychological, social, and economic well-being. As advances in early diagnosis and cancer treatment have significantly improved survival rates, increasing attention has been directed toward survivorship issues and the preservation of health-related quality of life (HRQoL) among oncology patients [1,2]. Beyond the biological burden of the disease, patients with cancer frequently experience persistent physical symptoms, including fatigue, pain, dyspnea, insomnia, appetite disturbances, and functional impairment, which negatively affect their daily functioning and overall quality of life [1]. In addition, cancer is commonly associated with considerable psychological distress, including depression, anxiety, stress, uncertainty, and fear of disease progression, all of which may compromise treatment adherence, coping ability, and psychosocial adjustment [3,4]. Social limitations, impaired interpersonal relationships, and reduced participation in social activities are also frequently reported among cancer survivors, further contributing to emotional burden and poorer functioning [5]. Moreover, financial toxicity resulting from long-term treatment costs, reduced work capacity, and healthcare expenditures has emerged as an important determinant of poorer HRQoL and psychosocial outcomes in patients with cancer [6]. Consequently, contemporary oncology care increasingly emphasizes a holistic and patient-centered approach that integrates symptom management, psychosocial support, and quality of life assessment as essential components of comprehensive cancer care and survivorship research [4,5,7,8].

The COVID-19 pandemic has profoundly disrupted cancer care worldwide and has significantly affected the psychological well-being and quality of life of patients with cancer. Public health measures implemented to control the spread of the virus, including lockdowns, social distancing, quarantine measures, and restrictions in healthcare access, resulted in major changes in oncology services and supportive care delivery. Previous studies have shown that reductions in in-person consultations, delays in cancer screening, diagnosis, and treatment, as well as interruptions in supportive care services, negatively affected both the physical and psychological health of oncology patients [9]. In particular, delayed treatments, fear of infection, uncertainty regarding disease management, and limited communication with healthcare professionals contributed to increased levels of anxiety, depression, stress, and fear of cancer recurrence among cancer patients during the pandemic [10,11]. Moreover, social distancing measures and prolonged isolation intensified feelings of loneliness, emotional distress, and social disconnection, further compromising patients’ mental health and quality of life [12,13]. At the same time, healthcare systems rapidly adopted telehealth and digital care interventions to maintain continuity of oncology care and psychosocial support. Although remote healthcare services provided important alternatives during the pandemic, several studies highlighted limitations related to technological barriers, reduced interpersonal interaction, unequal access to digital resources, and difficulties fully addressing patients’ supportive care needs through virtual communication alone [14,15]. Nevertheless, telehealth interventions were shown to facilitate continuity of care and partially alleviate the burden associated with reduced hospital access during the pandemic [16,17].

Despite the growing body of literature examining psychological distress, quality of life, and supportive care among patients with cancer, important gaps in knowledge remain. Existing studies have primarily focused on specific psychosocial outcomes or particular cancer populations, while relatively few investigations have simultaneously explored the relationships between depression, anxiety, stress, perceived social support, and quality of life within the context of the post-COVID-19 era. Recent evidence suggests that cancer patients continue to experience substantial psychological distress during treatment, with depression and anxiety prevalence remaining particularly high among vulnerable oncology populations, especially following delays in diagnosis, treatment disruptions, and pandemic-related challenges [18]. Moreover, perceived social support and coping strategies appear to play a significant protective role in psychological adjustment and emotional well-being among patients with cancer. Although the COVID-19 pandemic has highlighted the importance of mental health and psychosocial care, most available studies have focused on the general population or healthcare professionals, while evidence specifically addressing oncology patients remains comparatively limited [19]. In addition, there is a scarcity of data examining these psychosocial dimensions in Greek cancer populations during the post-pandemic period. Therefore, further research is needed to better understand the associations between psychological distress, social support, and quality of life among patients with cancer in the aftermath of the COVID-19 pandemic. The present study aims to address this gap by investigating these interrelationships in a sample of Greek oncology patients.

Therefore, the aim of the present study was to investigate levels of depression, anxiety, stress, quality of life, and perceived social support among patients with cancer during the post-COVID-19 period, as well as to explore the associations between psychological distress, social support, demographic characteristics, treatment-related factors, and quality of life outcomes.

## 2. Materials and Methods

### 2.1. Study Design

This study employed a cross-sectional descriptive design to investigate the associations between psychological distress, quality of life, and perceived social support among patients with cancer during the post-COVID-19 period. The study was conducted within the framework of the research project entitled “Anxiety, Depression, and Quality of Life in Patients with Cancer after the COVID-19 Pandemic,” organized by Kapa3 and the “Adult Cancer Patient Care” Research Laboratory of the International Hellenic University, Thessaloniki.

### 2.2. Participants

The study sample consisted of 162 adult individuals diagnosed with neoplastic disease who were receiving follow-up care or treatment during the post-COVID-19 period. Participants were recruited using a convenience sampling method from oncology-related healthcare settings and patient support networks. Eligibility criteria included: (a) age ≥ 18 years, (b) confirmed diagnosis of cancer, (c) ability to understand and communicate in Greek, and (d) willingness to participate in the study. Patients with severe cognitive impairment or inability to complete the questionnaire independently were excluded from participation. All participants provided informed consent prior to participation, and anonymity and confidentiality of the collected data were ensured throughout the study process. Participant recruitment and eligibility screening were conducted by members of the research team affiliated with KAPA3 and the collaborating academic institutions. All researchers involved in data collection had previous experience in clinical and health research and were familiar with the study protocol, eligibility criteria, and ethical procedures. Before data collection, the research team received standardized instructions regarding participant recruitment, informed consent procedures, and questionnaire administration to ensure consistency throughout the study. Questionnaires were reviewed for completeness before data analysis. Cases with substantial missing data were excluded, while occasional missing responses were handled using available case analysis for the corresponding variables.

### 2.3. Measurements

Data were collected using a structured questionnaire consisting of four sections: (a) demographic and clinical characteristics, (b) psychological distress assessment, (c) quality of life assessment, and (d) perceived social support assessment.

#### 2.3.1. Demographic and Clinical Characteristics

Participants provided information regarding age, gender, marital status, place of residence, educational level, smoking and alcohol consumption, type of cancer diagnosis, insurance status, occupational status, and treatment modalities (e.g., surgery, chemotherapy, radiotherapy, immunotherapy, hormone therapy, and targeted therapy).

#### 2.3.2. Depression, Anxiety, and Stress Scale-21 (DASS-21)

Psychological distress was assessed using the Depression Anxiety Stress Scale-21 (DASS-21), a widely used self-report instrument designed to evaluate symptoms of depression, anxiety, and stress. The scale consists of 21 items divided into three subscales: depression, anxiety, and stress, with seven items in each subscale. Responses are rated on a 4-point Likert scale ranging from 0 (“Did not apply to me at all”) to 3 (“Applied to me very much or most of the time”). Higher scores indicate greater psychological distress.

#### 2.3.3. European Organization for Research and Treatment of Cancer Quality of Life Questionnaire-Core 30 (EORTC QLQ-C30)

Quality of life was assessed using the EORTC QLQ-C30 (version 3.0), a cancer-specific instrument developed by the European Organization for Research and Treatment of Cancer. The questionnaire consists of 30 items evaluating global health status/quality of life, five functional domains (physical, role, emotional, cognitive, and social functioning), and symptom scales/items (fatigue, pain, nausea/vomiting, dyspnea, insomnia, appetite loss, constipation, diarrhea, and financial difficulties). Scores are linearly transformed to a 0–100 scale according to the EORTC scoring manual. Higher scores on functional scales and global health status indicate better functioning and quality of life, whereas higher scores on symptom scales indicate greater symptom burden.

#### 2.3.4. Perceived Social Support Scale

Perceived social support was assessed using a 12-item multidimensional social support scale evaluating support from family, friends, and significant others. Responses were rated on a 7-point Likert scale ranging from 1 (“Totally disagree”) to 7 (“Totally agree”).

### 2.4. Statistical Analysis

Statistical analysis was performed using the Statistical Package for the Social Sciences (SPSS), version 26.0 (IBM Corp., Armonk, NY, USA). Descriptive statistics were used to summarize demographic, clinical, psychological, quality of life, and social support variables. Continuous variables were expressed as means and standard deviations (SD), whereas categorical variables were presented as frequencies and percentages. The internal consistency reliability of the study instruments was evaluated using Cronbach’s alpha coefficient. Values greater than 0.70 were considered indicative of acceptable reliability.

Exploratory factor analysis using Principal Component Analysis (PCA) with Varimax rotation was conducted to assess the factorial structure of the perceived social support scale. Sampling adequacy was evaluated using the Kaiser–Meyer–Olkin (KMO) index, while Bartlett’s test of sphericity was used to determine the suitability of the data for factor analysis.

Comparisons between groups were performed using independent samples *t*-tests for dichotomous variables and one-way analysis of variance (ANOVA) for variables with more than two categories. Only statistically significant differences were reported in the Section 3.

Correlations between psychological distress, quality of life, and social support variables were assessed using Spearman’s rho correlation coefficient due to the non-normal distribution of several variables. Correlation coefficients were interpreted according to the strength and direction of the association.

All statistical tests were two-tailed, and the level of statistical significance was set at *p* < 0.05.

## 3. Results

### 3.1. Demographic and Clinical Characteristics of the Participants

A total of 162 patients with neoplastic disease participated in the study, with a mean age of 54.4 years (SD = 11.1) (Table 1). The majority of participants were female (74.1%), married (58.6%), and residents of urban areas (77.2%). Regarding educational level, most participants had completed higher education (43.8%), while 18.5% held a Master’s or PhD degree. In terms of health-related behaviors, 31.5% of participants were smokers and 17.3% reported alcohol consumption. Breast cancer was the most common type of malignancy (44.4%), followed by cancers of the digestive system and female genital organs (11.1% each). Regarding treatment modalities, most patients had undergone surgery (74.5%) and chemotherapy (69.6%), while nearly half had received radiotherapy (49.7%). In addition, 29.2% were receiving hormone therapy, 28.6% immunotherapy, and 13.7% targeted therapy.

### 3.2. Results on Psychological Status of Patients: Depression, Anxiety, and Stress

Regarding depressive symptoms, 32.1% of participants reported severe or extremely severe depression, whereas only 42.0% presented normal or mild levels of depression. These findings indicate that approximately one in three patients experienced clinically significant depressive symptomatology. In terms of anxiety, 40.1% of the participants reported severe or extremely severe anxiety levels, highlighting a substantial psychological burden within the study population. Only 40.1% of patients demonstrated normal or mild anxiety levels. Concerning stress, 29.0% of participants experienced severe or extremely severe stress, while 53.7% reported normal or mild stress levels (Table 2).

The DASS-21 scale demonstrated high levels of psychological distress among patients with cancer. The internal consistency reliability of the instrument was excellent, with Cronbach’s alpha coefficients ranging from 0.878 to 0.924 for the subscales and 0.956 for the total scale, indicating high reliability and stability of the measurements (Table 3).

### 3.3. Results on Quality of Life Assessment Among Participants

The overall quality of life of the participants, as assessed by the EORTC QLQ-C30 Global Health Status scale, demonstrated a moderate level of perceived health status and quality of life (Mean = 56.74, SD = 21.73) (Table 4). Regarding functional status, higher scores indicate better functioning. The highest mean scores were observed in cognitive functioning (Mean = 65.95, SD = 28.32) and physical functioning (Mean = 64.90, SD = 23.41), suggesting that patients maintained, to some extent, their cognitive and physical abilities. In contrast, lower scores were reported for social functioning (Mean = 49.18, SD = 33.89) and emotional functioning (Mean = 50.87, SD = 27.78), indicating greater impairment in social interactions and emotional well-being. With respect to symptom burden, higher scores reflect greater symptom severity. The most prominent symptoms reported by patients were fatigue (Mean = 57.48, SD = 29.63), insomnia (Mean = 52.47, SD = 36.55), financial difficulties (Mean = 47.94, SD = 35.62), and dyspnea (Mean = 44.86, SD = 32.88). Lower symptom scores were observed for nausea and vomiting (Mean = 12.45, SD = 24.45).

### 3.4. Results on Perceived Social Support Scale

Factor analysis of the perceived social support scale identified two major sources of social support: (a) support from family and relatives and (b) support from friends and significant others. The adequacy of the sample for factor analysis was considered excellent (KMO = 0.913), while Bartlett’s test of sphericity was statistically significant (*p* < 0.001), supporting the suitability of the data for factor extraction. The two-factor solution explained 83.1% of the total variance (Table 5). Participants reported higher levels of support from family and relatives (Mean = 5.33, SD = 1.70) compared to support from friends and significant others (Mean = 4.85, SD = 1.78).

### 3.5. Significant Correlations Between Quality of Life, Psychological Distress, and Perceived Social Support Among Participants

Strong negative correlations were observed between depression, anxiety, stress, and quality of life indicators (Table 6). Specifically, higher levels of depression were associated with poorer functional status (r = −0.699, *p* < 0.001) and lower global health status (r = −0.511, *p* < 0.001), while higher depressive symptoms were positively associated with increased symptom burden (r = 0.578, *p* < 0.001). Similarly, anxiety demonstrated significant negative correlations with global health status (r = −0.467, *p* < 0.001) and functional status (r = −0.635, *p* < 0.001), whereas it was positively correlated with symptom severity (r = 0.622, *p* < 0.001). Stress was also strongly associated with poorer functioning (r = −0.717, *p* < 0.001), lower global quality of life (r = −0.486, *p* < 0.001), and increased symptoms (r = 0.572, *p* < 0.001). These findings indicate that increased psychological distress is closely related to poorer daily functioning and greater symptom burden among patients with cancer.

Perceived social support from both family and friends was associated with better quality of life and lower psychological distress. Family support was positively associated with global health status (r = 0.196, *p* = 0.012) and negatively associated with depression (r = −0.210, *p* = 0.007) and stress (r = −0.268, *p* = 0.001). Support from friends and significant others demonstrated even stronger protective associations. Higher levels of friends’ support were significantly associated with better functional status (r = 0.330, *p* < 0.001) and global health status (r = 0.321, *p* < 0.001), as well as lower levels of depression (r = −0.359, *p* < 0.001), anxiety (r = −0.276, *p* < 0.001), stress (r = −0.339, *p* < 0.001), and symptom burden (r = −0.217, *p* = 0.005).

### 3.6. Results on Demographic and Treatment-Related Factors Associated with Quality of Life

Several demographic and treatment-related factors were significantly associated with quality of life outcomes among patients with cancer. Female participants reported significantly higher levels of insomnia compared with male participants (Mean = 56.1 vs. 42.1, *p* = 0.032), indicating poorer sleep quality among women. Participants with higher educational attainment demonstrated significantly better quality of life outcomes. Specifically, individuals holding a Bachelor’s degree or postgraduate qualifications reported higher global health status scores and better physical functioning compared with participants with lower educational levels (*p* = 0.030 and *p* = 0.025, respectively). Married participants demonstrated significantly better overall functional status and cognitive functioning compared with divorced participants and individuals belonging to other marital status categories. These findings may reflect the beneficial role of partner support in maintaining daily functioning and cognitive well-being. Smokers exhibited poorer emotional functioning and significantly higher symptom burden compared with non-smokers. More specifically, smokers reported higher levels of fatigue, dyspnea, and insomnia (all *p* < 0.05), suggesting that smoking may further aggravate physical and psychological difficulties in patients with cancer (Table 7).

Immunotherapy was associated with poorer role and social functioning, greater overall symptom burden, and increased appetite loss. Patients receiving immunotherapy reported significantly lower social functioning scores and more severe symptoms compared with those not receiving immunotherapy. In addition, radiotherapy was significantly associated with greater financial difficulties. Chemotherapy was also associated with financial burden, highlighting the socioeconomic impact of cancer treatment on patients’ daily lives (Table 8).

## 4. Discussion

The findings of the present study indicate that patients with cancer continue to experience substantial psychological and psychosocial burden during the post-COVID-19 period. High levels of anxiety, depression, and stress were observed among participants, while quality of life was reported as moderate to low, particularly in emotional and social functioning domains. Patients also experienced considerable symptom burden, with fatigue, insomnia, and financial difficulties emerging as the most prominent problems affecting daily life. Furthermore, psychological distress was strongly associated with poorer quality of life and increased symptom severity. At the same time, perceived social support, especially support from family members and friends, appeared to function as an important protective factor, contributing to better quality of life outcomes and lower levels of psychological distress among oncology patients.

### 4.1. Psychological Distress Among Patients with Cancer in the Post-COVID-19 Period

The present study demonstrated that patients with cancer continued to experience considerable levels of depression, anxiety, and stress during the post-COVID-19 period, suggesting that the psychological consequences of the pandemic extend well beyond the acute public health emergency. Although healthcare services have largely resumed normal operations, many cancer survivors continue to experience emotional distress associated with fear of disease recurrence, uncertainty regarding future health, persistent disruptions in social relationships, and the cumulative burden of cancer diagnosis and treatment. These findings indicate that the psychological impact of the pandemic has evolved into a longer-term survivorship challenge rather than resolving with the lifting of public health restrictions. These findings are consistent with previous studies reporting increased emotional burden, fear, and psychosocial difficulties among cancer patients during and after the COVID-19 pandemic [20,21,22]. The elevated levels of psychological distress observed in the present study may be explained by the long-term consequences of pandemic-related disruptions in oncology care, including delays in diagnosis and treatment, reduced access to healthcare services, social isolation, fear of infection, and uncertainty regarding disease progression [23,24]. In addition, changes in healthcare delivery, such as restrictions on hospital visits and the rapid transition to telehealth services, may have further contributed to emotional distress and feelings of vulnerability among patients with cancer [25,26]. Previous literature has similarly shown that cancer survivors experienced worsening mental health outcomes, including increased depression, anxiety, insomnia, and loneliness during the pandemic period [27,28,29]. From a clinical perspective, these findings underscore the importance of integrating routine psychosocial assessment into oncology practice. Early identification of psychological distress through standardized screening, combined with timely referral to psycho-oncology services and multidisciplinary supportive care, may improve emotional well-being, treatment adherence, and health-related quality of life. Given the persistent psychological burden observed in the post-pandemic era, survivorship care models should incorporate long-term mental health support as an essential component of comprehensive cancer care [30,31].

### 4.2. Quality of Life and Symptom Burden Among Oncology Patients

The present study demonstrated that patients with cancer experienced moderate to low quality of life, particularly in the domains of emotional and social functioning, while fatigue, insomnia, dyspnea, and financial difficulties emerged as the most burdensome symptoms affecting daily functioning. These findings are consistent with previous studies demonstrating that symptom burden is a major determinant of health-related quality of life in oncology populations and that both physical and psychosocial symptoms frequently coexist, creating a cumulative negative effect on patients’ overall well-being [1,32,33]. Fatigue and insomnia are among the most prevalent and persistent symptoms experienced by cancer survivors and have been consistently associated with impaired physical functioning, emotional distress, cognitive difficulties, and reduced participation in social and occupational activities. Similarly, dyspnea may limit functional independence and contribute to anxiety by increasing patients’ perceptions of physical vulnerability, particularly among individuals with advanced disease or treatment-related complications. Financial difficulties represent another important dimension of cancer survivorship, as the economic burden associated with treatment costs, reduced work capacity, and long-term healthcare needs may further exacerbate psychological distress and diminish quality of life [34,35,36]. Collectively, these findings support the growing recognition that symptom management should extend beyond the treatment of physical manifestations of cancer and adopt a comprehensive, patient-centered approach addressing emotional, social, and financial needs. Routine assessment of symptom burden, combined with multidisciplinary supportive care interventions, may substantially improve patients’ quality of life and facilitate better long-term survivorship outcomes.

### 4.3. Associations Between Psychological Distress and Quality of Life

An important finding of the present study was the strong association between psychological distress and poorer quality of life among oncology patients. Higher levels of depression, anxiety, and stress were linked to reduced functioning, lower global health status, and increased symptom burden. Similar findings have been reported in previous studies showing that psychological distress negatively affects emotional, social, and physical functioning in patients with cancer [33,37]. Furthermore, symptoms such as fatigue, pain, insomnia, and dyspnea appear to aggravate emotional burden and further impair patients’ daily functioning and well-being [7,38,39]. Previous research has also demonstrated that anxiety and depressive symptoms are significantly associated with lower health-related quality of life across different cancer populations [40,41]. These findings emphasize the clinical importance of integrating psychological assessment and supportive interventions into routine oncology care.

### 4.4. The Protective Role of Perceived Social Support

An important finding of the present study was the strong association between psychological distress and poorer quality of life among oncology patients. Participants reporting higher levels of depression, anxiety, and stress also demonstrated poorer functioning, lower global health status, and a greater symptom burden, highlighting the close interrelationship between psychological well-being and health-related quality of life. This association is consistent with previous research indicating that psychological distress adversely affects emotional, social, cognitive, and physical functioning throughout the cancer trajectory. Support from family members and friends appeared to be associated with lower levels of depression, anxiety, and stress, as well as better global health status and functional outcomes. Similar findings have been reported in previous studies demonstrating that higher perceived social support is related to improved emotional well-being, better quality of life, and reduced symptom burden among patients with cancer [42,43]. Previous research has also shown that social support may buffer the negative psychological effects of cancer by reducing anxiety and depressive symptoms and enhancing patients’ coping abilities [44,45]. Furthermore, psychosocial and supportive care interventions, including nurse-led and digital support programs, have been associated with improvements in anxiety, symptom management, and functional status in oncology populations [46,47]. From a clinical perspective, these findings reinforce the importance of routinely screening for psychological distress as part of comprehensive oncology care. Early identification of patients experiencing emotional difficulties, followed by timely psycho-oncology referral and multidisciplinary supportive interventions, may improve symptom management, enhance treatment adherence, and ultimately promote better health-related quality of life throughout the survivorship continuum [48].

### 4.5. Sociodemographic and Treatment-Related Factors Associated with Quality of Life

The present study further demonstrated that both sociodemographic and treatment-related factors were associated with variations in quality of life among oncology patients. Female patients appeared to report greater symptom burden, particularly regarding insomnia and emotional difficulties, a finding consistent with previous studies showing sex-related differences in symptom experience and quality of life outcomes [34,49]. Higher educational level was associated with better quality of life and functioning, possibly reflecting improved health literacy and coping abilities. In addition, marital status and social relationships appeared to influence emotional and social functioning, supporting previous evidence highlighting the protective role of interpersonal support in cancer adaptation [50]. Smoking and unhealthy lifestyle behaviors were also associated with greater symptom burden and poorer functioning [51].

Regarding treatment-related factors, chemotherapy, radiotherapy, and immunotherapy appeared to differentially affect patients’ quality of life and symptom experience. Previous studies have shown that chemotherapy is frequently associated with fatigue, insomnia, and financial burden, while radiotherapy may negatively affect emotional, cognitive, and social functioning, particularly during active treatment phases [52,53]. At the same time, recent evidence suggests that immunotherapy-based approaches may maintain or improve health-related quality of life in some oncology populations despite treatment-related adverse effects [54,55]. Financial difficulties also emerged as an important factor associated with poorer emotional well-being and functioning, emphasizing the growing impact of financial toxicity in cancer care [56,57].

### 4.6. Clinical and Practical Implications

The findings of the present study have important clinical implications for oncology care. Given the high levels of depression, anxiety, stress, and symptom burden identified among patients with cancer, routine psychological screening should be systematically integrated into clinical practice in order to facilitate early identification of patients at risk for psychological distress and poor quality of life. The integration of psycho-oncology services within standard cancer care may contribute to improved emotional well-being, symptom management, and treatment adaptation [41,47]. In addition, the strong association between perceived social support and better quality of life highlights the importance of multidisciplinary survivorship care approaches that include psychosocial support interventions involving healthcare professionals, family members, and caregivers [42,43]. Previous studies have also demonstrated that supportive care strategies, including nurse-led interventions, rehabilitation programs, and telehealth or digital support services, may reduce distress, improve symptom management, and enhance functional outcomes among oncology patients [46,58,59]. Therefore, comprehensive and patient-centered supportive care models should be prioritized to address the multidimensional needs of cancer survivors and improve their overall quality of life. Beyond its immediate effects on healthcare delivery, the COVID-19 pandemic has substantially influenced the perspectives of cancer survivors, healthcare professionals, and policymakers regarding supportive oncology care. For many cancer survivors, the pandemic highlighted the importance of psychological resilience, social support, and timely access to reliable healthcare services as essential components of survivorship. Likewise, clinicians have increasingly recognized that optimal cancer care extends beyond disease management to include routine psychosocial assessment, mental health support, and patient-centered communication. The rapid implementation of telehealth during the pandemic further demonstrated its potential to improve access to supportive care for selected patients, although it also underscored existing inequalities related to digital literacy and healthcare accessibility. At the health system level, the pandemic emphasized the need for more resilient oncology services capable of maintaining continuity of cancer care during future public health crises. These lessons should inform future survivorship care models and healthcare policies by promoting integrated multidisciplinary care, strengthening psychosocial support services, and ensuring equitable access to oncology care for all patients.

Furthermore, the findings of this study support the implementation of multidisciplinary rehabilitation programs that integrate medical, nursing, psychological, physical, and social support services to address the complex needs of cancer survivors. Routine screening for psychological distress and symptom burden should be incorporated into standard oncology practice to facilitate early identification of patients requiring supportive interventions. These findings may also contribute to the development of evidence-based clinical guidelines and public health policies that promote comprehensive survivorship care, strengthen psychosocial support services, and improve long-term health outcomes and quality of life among oncology patients.

### 4.7. Strengths and Limitations of the Study

The present study has several strengths that should be acknowledged. First, validated psychometric instruments were used to assess psychological distress, perceived social support, and quality of life, enhancing the reliability and validity of the findings. In addition, the study adopted a multidimensional approach by simultaneously examining psychological, social, and functional aspects of patients’ experiences. Another important strength is the investigation of oncology patients within the post-COVID-19 context, an area that remains relatively underexplored in current literature. Furthermore, the inclusion of a Greek oncology population contributes valuable data to the limited evidence available from Greece regarding the psychosocial impact of cancer and survivorship.

However, several limitations should be considered when interpreting the findings. The relatively small sample size and the recruitment of participants from a single national patient support network using convenience sampling may limit the generalizability of the findings to the broader oncology population. In addition, convenience sampling may limit the generalizability of the findings to the broader oncology population. In addition, because participants were recruited using a convenience sampling approach, detailed information regarding the number of individuals initially approached, excluded, or declining participation was not systematically recorded, precluding the presentation of a participant flow diagram. The use of self-reported questionnaires may also have introduced response and recall bias. Furthermore, the predominance of women and breast cancer patients in the sample may have influenced the overall findings and reduced representativeness across different cancer types. Future longitudinal, multicenter studies involving larger and more heterogeneous samples across different cancer types are warranted to confirm the present findings and improve their generalizability.

## 5. Conclusions

Cancer patients in the post-COVID-19 era continue to experience considerable psychological distress and impaired quality of life, with anxiety, depression, stress, fatigue, insomnia, and financial difficulties remaining major challenges in daily living. At the same time, social support from family and friends appears to significantly alleviate emotional burdens and promote better functioning and well-being. The findings of the present study reinforce the urgent need for comprehensive oncology care models that address not only the physical aspects of cancer but also the psychological and social dimensions of survivorship.

## Figures and Tables

**Table 1:** Demographic and clinical characteristics of the study sample.

Variable	*n*	%
Gender		
Female	120	74.1
Male	42	25.9
Marital status		
Married	95	58.6
Single	35	21.6
Divorced	21	13.0
Cohabitation	5	3.1
Widowed	6	3.7
Place of residence		
Urban area	125	77.2
Semi-urban area	20	12.3
Rural area	17	10.5
Educational level		
Primary school	7	4.3
Secondary school	10	6.2
High school graduate	44	27.2
Higher education	71	43.8
Master’s degree	25	15.4
PhD degree	5	3.1
Smoking status		
Yes	51	31.5
No	111	68.5
Alcohol consumption		
Yes	28	17.3
No	134	82.7
Cancer type (ICD-10)		
Breast cancer	72	44.4
Digestive system cancers	18	11.1
Female genital organ cancers	18	11.1
Respiratory system cancers	13	8.0
Hematologic/lymphatic cancers	11	6.8
Multiple primary cancers	9	5.6
Male genital organ cancers	7	4.3
Other cancer types	14	8.7
Treatment modalities *		
Surgery	120	74.5
Chemotherapy	112	69.6
Radiotherapy	80	49.7
Hormone therapy	47	29.2
Immunotherapy	46	28.6
Targeted therapy	22	13.7

* Participants could receive more than one treatment modality.

**Table 2:** Distribution of DASS-21 severity levels among participants.

DASS-21 Scale	Severity Level	*n*	%
Depression	Normal	44	27.2
	Mild	24	14.8
	Moderate	42	25.9
	Severe	15	9.3
	Extremely severe	37	22.8
Anxiety	Normal	60	37.0
	Mild	5	3.1
	Moderate	32	19.8
	Severe	21	13.0
	Extremely severe	44	27.2
Stress	Normal	66	40.7
	Mild	21	13.0
	Moderate	28	17.3
	Severe	25	15.4
	Extremely severe	22	13.6

**Table 3:** Reliability and descriptive statistics of the DASS-21 scales.

Scale	Cronbach’s α	Mean	SD
Depression	0.908	8.48	5.98
Anxiety	0.878	6.63	5.47
Stress	0.924	9.30	5.81
Total DASS-21	0.956	—	—

**Table 4:** EORTC QLQ-C30 quality of life scores among participants.

Scale	Mean	SD
Global Health Status/QoL	56.74	21.73
Functional Scales		
Physical functioning	64.90	23.41
Role functioning	59.36	34.25
Emotional functioning	50.87	27.78
Cognitive functioning	65.95	28.32
Social functioning	49.18	33.89
Symptom Scales		
Fatigue	57.48	29.63
Nausea and vomiting	12.45	24.45
Pain	34.98	30.19
Dyspnea	44.86	32.88
Insomnia	52.47	36.55
Appetite loss	23.05	32.02
Constipation	27.16	32.44
Diarrhea	23.87	31.41
Financial difficulties	47.94	35.62

**Table 5:** Factor analysis and descriptive statistics of perceived social support.

Variable	Value
Kaiser–Meyer–Olkin (KMO)	0.913
Bartlett’s Test of Sphericity	*p* < 0.001
Number of extracted factors	2
Total explained variance	83.1%

**Table 6:** Significant correlations between quality of life, psychological distress, and social support.

Variables	r	*p*-Value
Psychological distress and quality of life		
Depression—Global Health Status	−0.511	<0.001
Depression—Functional status	−0.699	<0.001
Depression—Symptoms	0.578	<0.001
Anxiety—Global Health Status	−0.467	<0.001
Anxiety—Functional status	−0.635	<0.001
Anxiety—Symptoms	0.622	<0.001
Stress—Global Health Status	−0.486	<0.001
Stress—Functional status	−0.717	<0.001
Stress—Symptoms	0.572	<0.001
Social support and quality of life		
Family support—Global Health Status	0.196	0.012
Family support—Depression	−0.210	0.007
Family support—Stress	−0.268	0.001
Friends support—Global Health Status	0.321	<0.001
Friends support—Functional status	0.330	<0.001
Friends support—Symptoms	−0.217	0.005
Friends support—Depression	−0.359	<0.001
Friends support—Anxiety	−0.276	<0.001
Friends support—Stress	−0.339	<0.001

**Table 7:** Significant associations between demographic factors and EORTC QLQ-C30 scores.

Variable	Outcome	Group Comparison	Mean ± SD	*p*-Value
Gender	Insomnia	Female	56.1 ± 36.9	0.032
		Male	42.1 ± 33.8	
Educational level	Global Health Status	Master’s/PhD	60.0 ± 18.1	0.030
		Bachelor degree	60.8 ± 23.5	
		High school graduate	49.8 ± 18.6	
		Up to secondary education	52.0 ± 23.9	
	Physical functioning	Master’s/PhD	69.3 ± 20.8	0.025
		Bachelor degree	69.1 ± 23.0	
		High school graduate	58.5 ± 22.5	
		Up to secondary education	56.1 ± 27.3	
Marital status	Functional status	Married	61.2 ± 21.5	0.022
		Single	60.0 ± 18.6	
		Divorced	53.3 ± 24.4	
		Other	40.0 ± 22.0	
	Cognitive functioning	Married	69.3 ± 26.2	0.006
		Single	71.4 ± 25.7	
		Divorced	54.0 ± 34.1	
		Other	42.4 ± 27.2	

**Table 8:** Significant associations between smoking, treatment modalities, and EORTC QLQ-C30 scores.

Variable	Outcome	Group Comparison	Mean ± SD	*p*-Value
Smoking	Emotional functioning	Non-smokers	54.4 ± 28.3	0.016
		Smokers	43.3 ± 25.3	
	Total symptom score	Non-smokers	35.5 ± 21.1	0.015
		Smokers	41.6 ± 16.5	
	Fatigue	Non-smokers	53.9 ± 30.6	0.020
		Smokers	65.4 ± 26.1	
	Dyspnea	Non-smokers	40.8 ± 34.4	0.013
		Smokers	53.6 ± 27.6	
	Insomnia	Non-smokers	48.3 ± 36.4	0.035
		Smokers	61.4 ± 35.5	
Immunotherapy	Role functioning	No	62.8 ± 33.6	0.039
		Yes	50.7 ± 34.6	
	Social functioning	No	53.9 ± 34.0	0.005
		Yes	37.3 ± 30.9	
	Total symptom score	No	35.4 ± 19.7	0.034
		Yes	42.5 ± 19.6	
	Appetite loss	No	19.3 ± 30.2	0.015
		Yes	32.6 ± 34.8	
Radiotherapy	Financial difficulties	No	41.1 ± 35.6	0.011
		Yes	55.0 ± 34.4	
Chemotherapy	Financial difficulties	No	57.7 ± 36.0	0.011
		Yes	45.4 ± 32.4	

## Data Availability

Data available upon reasonable request.
